# Dilatation of the Urethra by the Urine Itself

**Published:** 1878-02

**Authors:** 


### Dilatation of the Urethra by the Urine itself.

Towards the end of the last century Brunninghousen recommended this method of dilatation, which he claimed to be more easy and simple than that by bougies. To practice it the patient must simply compress lightly the urethra behind the glands with his fingers whenever he wishes to urinate. The pressure must be such that the urine can only escape slowly and after having remained some time in the canal; as a necessary result the canal will be more or less dilated through its entire length, in the constricted as well as in the healthy portion. If this be repeated every time the urine is voided, the same effects will gradually be produced as if bougies had been used, while at the same time the inconveniences of the latter are avoided. M. Bérenger-Férand has employed this method in his practice, and the following are the conclusions he has arrived at with regard to it:

1. Dilatation of the urethra by the urine, repeated at each urination for a long time after a prolonged attack of gonorrhœa, seems to prevent the formation of strictures.

2. In cases of moderate strictures it seems to have restored the normal calibre of the canal, or at least to have restored the calibre sufficiently to render micturition easy.

3. After the operation of urethrotomy it will perhaps prove useful to prevent, or at least to retard notably, the return of the constriction.

4. In cases of varicose dilatations at the neck of the bladder, or in the membranous portion of the urethra, it appears calculated to be serviceable.

5. It seems to prove useful also in the case of partial or total hypertrophy of the prostate in old men. In such patients the first drops of urine, which are emitted with so much difficulty and slowness, will serve effectually to fill the canal if the meatus be kept closed. When the ordinary calibre of the canal is once re-established in this way, the remaining contents of the bladder can be evacuated easily. The method has this great advantage, that it does away with the difficulty of emission after the first drops have escaped from the bladder; when it is not employed, the difficulty of emission persists during the entire act; the micturition, moreover, becomes intermittent and the bladder is incompletely emptied, as a result of which, frequent desire to urinate is soon experienced.—*Lyon Medi al Record,* October 7, 1877.

